# The *Pochonia chlamydosporia* Serine Protease Gene *vcp1* Is Subject to Regulation by Carbon, Nitrogen and pH: Implications for Nematode Biocontrol

**DOI:** 10.1371/journal.pone.0035657

**Published:** 2012-04-27

**Authors:** Elaine Ward, Brian R. Kerry, Rosa H. Manzanilla-López, Gerald Mutua, Jean Devonshire, John Kimenju, Penny R. Hirsch

**Affiliations:** 1 Plant Pathology and Microbiology Department, Rothamsted Research, Harpenden, Herts, United Kingdom; 2 Department of Plant Science and Crop Protection, University of Nairobi, Nairobi, Kenya; Kyushu Institute of Technology, Japan

## Abstract

The alkaline serine protease VCP1 of the fungus *Pochonia chlamydosporia* belongs to a family of subtilisin-like enzymes that are involved in infection of nematode and insect hosts. It is involved early in the infection process, removing the outer proteinaceous vitelline membrane of nematode eggs. Little is known about the regulation of this gene, even though an understanding of how nutrients and other factors affect its expression is critical for ensuring its efficacy as a biocontrol agent. This paper provides new information on the regulation of *vcp1* expression. Sequence analysis of the upstream regulatory region of this gene in 30 isolates revealed that it was highly conserved and contained sequence motifs characteristic of genes that are subject to carbon, nitrogen and pH-regulation. Expression studies, monitoring enzyme activity and mRNA, confirmed that these factors affect VCP1 production. As expected, glucose reduced VCP1 expression and for a few hours so did ammonium chloride. Surprisingly, however, by 24 h VCP1 levels were increased in the presence of ammonium chloride for most isolates. Ambient pH also regulated VCP1 expression, with most isolates producing more VCP1 under alkaline conditions. There were some differences in the response of one isolate with a distinctive upstream sequence including a variant regulatory-motif profile. Cryo-scanning electron microscopy studies indicated that the presence of nematode eggs stimulates VCP1 production by *P. chlamydosporia*, but only where the two are in close contact. Overall, the results indicate that readily-metabolisable carbon sources and unfavourable pH in the rhizosphere/egg-mass environment may compromise nematode parasitism by *P. chlamydosporia*. However, contrary to previous indications using other nematophagous and entomopathogenic fungi, ammonium nitrate (e.g. from fertilizers) may enhance biocontrol potential in some circumstances.

## Introduction

Root-knot nematodes (RKN, *Meloidogyne* spp.) are a major constraint to crop production worldwide, but particularly in the tropics. Infestation with RKN reduces the efficiency of roots at withdrawing nutrients and water from soil, sometimes causing the total failure of crops grown by resource-poor farmers in developing countries. Nematicides are some of the most toxic products used in crop protection and they have been banned in several European countries due to environmental concerns. They are also inappropriate or too expensive for use in many situations, so there is an urgent need for new methods of nematode management.

The fungus *Pochonia chlamydosporia* (previously *Verticillilum chlamydosporium*, teleomorph *Metacordyceps chlamydosporia* [1], [2], [3]) is a promising biological control agent for root-knot and cyst nematodes [4], [5], [6]. It colonizes the surface of plant roots and switches to become a parasite on contact with nematode egg masses produced by *Meloidogyne* spp., destroying the eggs. There is no simple relationship between fungal abundance in soil and parasitic activity, which is significantly affected by nutrition [4]. Effective establishment of microbes applied to soil often requires addition of an exogenous nutrient source to overcome competition from the resident microflora. However, readily available nutrients may compromise the parasitic activity of microbial facultative parasites added as biological control agents. More information is therefore needed on nutritional and environmental factors that affect the efficacy of biological control agents such as *P. chlamydosporia*.

This study focuses on the regulation of VCP1, an alkaline serine protease that is important in the early stages of nematode egg infection, at the onset of the parasitic phase. This subtilisin-like enzyme removes the outer proteinaceous vitelline membrane of the egg, allowing hyphae to penetrate and infect [7], [8]. The aim of this work was to study the effect of nutrients and pH on expression of the VCP1 gene. The upstream region of the gene was sequenced in 30 *P. chlamydosporia* isolates to determine any variation in putative regulatory motifs. Selected isolates were then used to examine the effect of glucose, ammonium chloride and pH on VCP1 production in the presence and absence of *M. incognita* eggs. Electron microscopy was used to investigate any damage to the eggs caused by the fungus in the different growth media. Implications of the findings for optimising the conditions for application of *P. chlamydosporia* as a biocontrol agent are discussed.

## Materials and Methods

### Fungal and Nematode Cultures

A population of root-knot nematode (RKN) *Meloidogyne incognita* was maintained and reproduced on susceptible tomato plants (*Solanum lycopersicum* cv. Tiny Tim) in a glasshouse at 24–26°C. Nematode cultures were started from a single egg mass. Three-week-old tomato seedlings were transplanted in sterile compost and inoculated with freshly hatched surface sterilised second-stage juveniles (J2). Roots infected by mature females were washed gently to eliminate soil and plant debris prior to removing egg masses with fine forceps. Egg masses were rinsed in sterile distilled water (sdH_2_O) and eggs were released using bleach (0.3%) and washed four times in sdH_2_O. Sterility of the eggs was checked by plating on water agar (0.05%) plates incubated at 25°C for 1 week.

Isolates of *P. chlamydosporia* (Table 1) were obtained as freeze dried cultures from the Rothamsted culture collection. All the isolates were confirmed to be *P. chlamydosporia* using diagnostic PCR [9] and/or ITS sequencing. The fungi were grown on potato dextrose agar (Oxoid) and incubated at 28°C for 7–14 days. Conidia were harvested from the plates by pouring 5 ml of sterile 15% glycerol onto each plate followed by gentle scraping of the fungal colonies with a plate spreader. The liquid was then filtered through Miracloth, and stored in aliquots at –80°C until needed.

**Table 1:** *Pochonia chlamydosporia* isolates used.

Isolate	Synonym	Host nematode	Country of origin
Pc10	IMI 331547	*M. incognita*	Brazil
Pc104		*Heterodera schachtii*	UK
Pc133	13t3	RKN^1^	Kenya
Pc147	23a	*M. incognita*	Kenya
Pc190	(Kenya 2) 24	RKN^1^	Kenya
Pc280	VCJ, IMI 380407	*Globodera rostochiensis*	Jersey
Pc392^2^	IMI SD 187	*M. incognita*	Cuba
Pc400	CMA	*Meloidogyne sp.*	Bulgaria

1 The source location indicates a root-knot nematode host [61].

2 A P. *chlamydosporia* var. *catenulata* strain.

For the gene expression studies approximately 1×10^4^ conidia were inoculated into 150 ml of supplemented Czapek Dox broth (NaNO_3_ 3 g, KCl 0.5 g, magnesium glycerophosphate 0.5g, FeSO_4_ 0.01 g, K2SO_4_ 0.35 g, sucrose 30 g, 0.5 g yeast extract, pH 6.8) l^–1^ in 250 ml conical flasks and incubated at 28°C for 3 days with constant shaking at 200 rpm. The resulting mycelium was harvested and washed in sterile distilled water before transferring to flasks containing 25 ml of 0.01M K phosphate buffer (pH 6.8) (abbreviated to P in this study) or P containing one or more of the following additives: 2% D-glucose, 200 mM NH_4_Cl, *M. incognita* eggs (1 egg µl^–1^). To investigate the effect of pH, 0.01M K phosphate buffers at pH 5.8 and pH 8.0 were used. Flasks (three biological reps per treatment) were incubated at 28°C with shaking at 130 rpm and two 1.9 ml samples were taken at 0, 4, 8 and 24 h. After centrifugation at 16.1g in a microfuge, the supernatant was removed and stored at 4°C until enzyme assays were completed (within 24 h). The fungal pellets were immediately frozen in liquid nitrogen and stored at –80°C before being used to extract RNA.

### Extraction and Purification of RNA and DNA

DNA was prepared from freeze-dried mycelium from liquid cultures of *P. chlamydosporia* as described in [10]. RNA was extracted from aliquots of approximately 100mg frozen fungal mycelia. This was added to Lysing matrix C (Bio101) and 1 ml Trizol reagent (Invitrogen) in a 2 ml screw top microtube and shaken in a FastPrep® cell disrupter (Thermo-Savant) at 6 ms^–1^ for 45 sec. The Invitrogen protocol for RNA extraction using Trizol reagent (https://tools.invitrogen.com/content/sfs/manuals/15596018.pdf) was then followed except that BCP phase separation reagent (Helena biosciences) was used instead of chloroform. Residual DNA was removed using the Turbo DNA-free kit (Ambion). The resulting RNA preps were tested (without reverse transcription) in VCP1-specific PCRs (see below) to ensure that they did not contain DNA.

### Amplification, Sequencing and Analysis of the Upstream Region of *vcp1*

Initially a two-step magnetic bead capture method [11] was used to isolate the upstream flanking regions of the VCP1 gene of Pc10, 104, 190, 280 and 400. The sequences of the primers used are given in Table 2. VCP1 gene specific primers (VCP1 F2rev [biotinylated] and VCP1 SFrev) were used in combination with partly degenerate primers (FP1-4) and a non-degenerate primer contained within these (FSP [biotinylated]). The biotin-labelled VCP1 flanking regions were isolated using a streptavidin-magnetic bead capture protocol. Several bands with sizes around 800bp were cloned into pGEM-T-Easy® (Promega) and sequenced at the Geneservice sequencing facility (http://www.geneservice.co.uk). DNA sequences were assembled using the STADEN package (Medical Research Package, Laboratory of Molecular Biology, Cambridge UK). An alignment of these sequences was generated using ClustalX v2.0 [12]. This was used to design a new PCR assay (using primers VCPuff5 and VCP1 SFrev) which was then used to amplify specifically the upstream region of *vcp1* in other isolates. These were then sequenced directly, or after cloning, using VCP1 uff5, VCP1 uff1, VCP1 SFrev and M13 primers. Vector NTi (Invitrogen) was used to determine the location of nucleotide motifs on the sequences.

**Table 2:** Primers and probes used.

Name	sequence 5'–3'
FP1	CAGTTCAAGCTTGTCCAGGAATTCNNNNNNNGGCCT
FP2	CAGTTCAAGCTTGTCCAGGAATTCNNNNNNNGCGCT
FP3	CAGTTCAAGCTTGTCCAGGAATTCNNNNNNNCCGGT
FP4	CAGTTCAAGCTTGTCCAGGAATTCNNNNNNNCGCGT
FSP	CAGTTCAAGCTTGTCCAGGAATTC
VCP1 F2rev	CCA GTG TCA AGG ACG TAG ACA CAA G
VCP1 SFrev	AGA AGA ACA GAC AGT TGC AT
VCP1uff1	TCTGGACTATGGCGATCATGGT
VCP1 uff5	CCGGTTTGGACTTTGCAGCG
btubqrnaF2	TCCCTCGTCTGCACTTCTTCA
btubqrnaR1	CCATTCGACAAAGTAGGTCGAGTT
btubqrnaP2	TGACCTGCTCTGCCATCTTCCGTG
vcp1qrnaF1	GTCTACAGCCACCTCTTCAATGG
vcp1qrnaR1	GGTGGTGCTTCCCTTTTGG
vcp1qrnaP1	CACCCAGATGTTGACTTCATCGAGAAGGAC

### VCP1 Enzyme Assays

VCP1 activity was assayed using N-Succinyl-Ala-Ala-Pro-Phe p-nitroanilide (Sigma) by adapting a previously used method [7] for high throughput analysis. Culture supernatant (10 µl), substrate (100 µl of 2 mM) and buffer (90 µl of 0.1M Tris-HCl pH 7.9) were mixed in a flat bottom microwell plate (Nunc) and the absorbance at 410 nm was measured every 8 sec for 3 min at 25°C using a Varioskan (Thermo Electron Corp) plate reader. There were three technical reps for each sample. Enzyme activity was expressed as the value of the increase in absorbance per min divided by the weight of fungus in the 3.8 ml of culture sampled.

### Quantitative Real-time PCR

Previously published sequences of the VCP1 and beta-tubulin genes of *P. chlamydosporia* isolates [13] were aligned to identify suitable regions for PCR assay design. Primers and probes (Table 2) were then chosen using Primer Express software (Applied Biosystems). Assays (25 µl) to detect cDNA from VCP1 and beta-tubulin were performed using Jump Start Ready mix for quantitative PCR (Sigma). Primer, probe and MgCl_2_ concentrations were optimized experimentally according to the manufacturer’s guide. For *vcp1* amplification 400 nM vcp1qrnaF1 primer, 400 nM vcp1qrnaR1 primer and 200 nM probe vcp1qrnaP1 were used. For beta-tubulin gene amplification 400 nM btubqrnaF2 primer, 600 nM btubqrnaR1 primer and 150 nM probe btubqrnaP2 were used. Dual labelled probes (www.biomers.net) were labelled at the 5′ end with FAM (6-carboxy-fluoroscein) and at the 3′ end with a dark hole quencher. For both assays MgCl_2_ was added to give a final concentration of 5 mM. In all cases 2 µl of a 1/5 dilution of a cDNA prep (generated using the Superscript III First Strand Synthesis System for RT PCR [Invitrogen]) from 500 ng RNA was added to the PCR. Amplification and detection were performed in an ABI Prism 7500 Sequence Detection System (Applied Biosystems) as follows: one cycle of 94°C for 2 min followed by 40 cycles of 94°C 15sec, 60° 1 min. The VCP1 mRNA levels were calculated from three technical reps, by the ABI software, as normalized relative quantification (NRQ) values using the 2^–ΔΔCT^ method [14] with beta-tubulin as the endogenous control and gene expression levels in the corresponding un-amended phosphate buffer (pH 6.8) medium at time point zero as calibrator.

### Statistical Analyses

Analysis of variance (ANOVA) was applied to the enzyme assay data (using Genstat ® [2011], fourteenth edition, ©VSN International Ltd., Hemel Hempsted, UK) to consider the main effects and interactions between different medium amendments and time points. The data were checked for conformation to a Normal distribution and to ensure the homogeneity of variance by plotting histograms of residuals and plotting the residuals against the fitted values. Where data showed a clear skewed distribution they were log (to base e) transformed, adding a small adjustment where necessary to account for zero observations (half the lowest positive value observed). Following ANOVA least significant differences (LSD) were used to statistically separate the means at the 5% level of significance. ANOVA was also used in a similar way to analyse the real-time PCR data, but this time using a transformation of the normalised relative quantity (NRQ) values as log_2_(1/NRQ) As the efficiency of all reactions were always close to optimal i.e. with value 2, this transformation returns values equivalent to the ΔΔC_T_ values calculated by the ABI software.

### Cryo-Scanning Electron Microscopy (CSEM)

Individual mycelium masses, (approximately 4 mm diameter) grown for 24h in different culture media together with nematode eggs, were carefully removed from the flasks. They were placed in 1.5 ml microtubes containing 1 ml of 1% glutaradehyde in phosphate buffer pH 7.2 to fix the samples. After 4–16 h at 4°C, the samples were gently washed 3 times in phosphate buffer with a final rinse in dH_2_O. Just prior to cryo-preparation, each sample was examined in a glass Petri dish under a stereo microscope to confirm that nematode eggs were present in the mycelium masses. A single mass containing eggs was picked up using a sterile syringe and placed directly onto a 10 mm diameter filter paper attached to the CSEM stub with a smear of OCT compound (Agar Scientific). Each sample was quickly plunged into pre-frozen liquid nitrogen and transferred under vacuum to the cold stage (–150^o^C) of the Alto 2500 cryo-chamber (Gatan) attached to the scanning electron microscope (SEM). Any contaminating ice was removed from the sample through sublimation by raising the temperature of the stage from –150^o^C to –95^o^C for 2 min. The temperature was returned to –150^o^C at which point the sample was coated with AuPd for 60 s. The sample was then transferred to the pre-cooled stage (–150^o^C) in the JSM 6700FEG Field Emission SEM (Jeol) for imaging. An anti-contaminator mounted just above the specimen stage was maintained at –170^o^C to keep the sample surface free of artefacts. An accelerating voltage of 3kV was used with a working distance of 5–6 mm. All micrographs were captured and saved using the on-board digital imaging software (Jeol).

## Results

### The Upstream Region of *vcp1* is Highly Conserved in *P. chlamydosporia* Isolates and Contains Motifs Characteristic of Carbon, Nitrogen and pH Regulation

**Figure 1:**
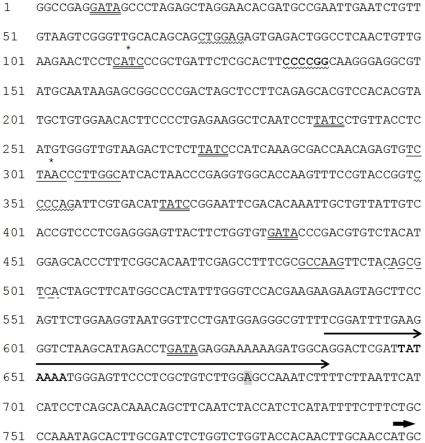
Sequence of the upstream region of the *vcp*1 gene of Pc400. This corresponds to nucleotides 214–1012 of the sequence deposited in EMBL/Genbank (Accession no. HE653898). The promoter region and ATG start codon are marked by narrow and thick arrows respectively and the transcription start site is shaded. The position of putative regulatory motifs in Pc400 or (where asterisked) other isolates (see Table 3) are underlined as follows: double line = GATA; wavy = CREA; dashed = CREB; single = PacC.

Initially a method with a combination of random and gene-specific primers [11] was used to isolate the upstream flanking regions of *vcp1* from isolates Pc10, 104, 190, 280 and 400 which were then sequenced. An alignment of these sequences was used to design new specific PCR primers (VCPuff5 and VCP1 SFrev) which were then used to amplify and sequence the region of a further 22 isolates. The sequences of this region in isolates Pc10, 400 (Fig. 1), 147, 280, 392, 104 and 133 have been deposited in EMBL/Genbank under the accession numbers HE653897 to HE653903.

**Table 3:** Positions of putative regulatory motifs upstream of the VCP1 coding region in *P. chlamydosporia* isolates.

Isolate	CREA			CREB	GATA							PacC			Promoter
Position on Fig. 1	72	133*	350*	497	8	111*	238*	272*	367*	430	620	300*	307*	486	640–688
Distance upstream	609	548	331	184	673	570	443	409	314	251	61	381	374	195	
Pc10	Y	Y	Y	Y	Y	N	Y	Y	Y	Y	Y	N	Y	Y	Y
Pc280	Y	Y	Y	Y	Y	Y	Y	Y	Y	Y	Y	N	Y	Y	Y
Pc133	Y	Y	Y	Y	Y	Y	Y	Y	Y	Y	Y	N	Y	Y	N
Pc147	Y	Y	Y	Y	Y	Y	Y	Y	Y	Y	Y	N	Y	Y	Y
Pc392	Y	Y	Y	Y	Y	N	Y	Y	N	Y	N	Y	Y	Y	N

The sequences of these motifs (5′ to 3′) are as follows: CREA = SYGGRG; CREB = CAGCGTCA; GATA = GATA; PacC = GCCARG, where S = C/G, Y = C/T and R = G/A. Positions given correspond to the 5′ end of the motif except where the motif is on the complementary strand (*) when the 3′end is shown. Distance upstream indicates the distance from the transcript start. Y indicates the sequence conforms to the consensus, N that it does not.

All the 30 isolates sequenced were≥97% identical to one another in this region with the exception of Pc392 (the P. *chlamydosporia* var. *catenulata* strain) which was 91–92% identical to the others. Five other isolates, including Pc133 and 280, had unique sequences. The most commonly found sequence types were typified by isolate Pc10 (identical to six others) and Pc147 (identical to Pc190 and six others). Some of the isolates (including Pc104 and 280) had a deletion of 5bp (corresponding to nucleotides (nt) 385–389 on the Pc10 sequence in Fig. 1) compared to the other isolates.

Positions of the putative regulatory motifs CREA and CREB (C-repression), GATA (N-repression) and PacC (pH regulation) were determined using VectorNTi and these are shown in Figure 1 for Pc400 and Table 3 for the isolates later used in the VCP1 expression studies (Pc10, 280, 133, 147 and 392). The position of the transcription start and promoter region had been predicted earlier for Pc10 [15]. In all isolates sequenced except Pc392, the presence/absence profiles for all the motifs were identical except for the putative GATA site 570 nt upstream of the transcription start. This site was present in Pc280, 133, 147 and 104 but was absent in Pc10 and 392. Isolate Pc392 shared the same CREA and CREB motif profiles as the other isolates but was unique in lacking the putative GATA sites at 314 and 61 nt upstream of the transcription start. It also had an extra putative PacC site 381 nt upstream of the transcription start that was not present in any other isolate sequenced. There was also one nt difference in the promoter region of Pc392 (nt 678 A to G, on Fig. 1) compared to other isolates and Pc133 had two differences here from all other isolates namely a C to T substitution at nt 664 and a T to C substitution at nt 671.

### The Presence of Glucose in Growth Medium Represses VCP1 Gene Expression

Following the VCP1 upstream region analysis, representatives of the two most common sequence types (Pc10 and 147) and three of the unique sequence types (Pc133, 280 and 392) were chosen for the VCP1 expression studies. In all experiments the enzyme levels were very low (close to the threshold of detection) at 4 h and earlier.

**Figure 2:**
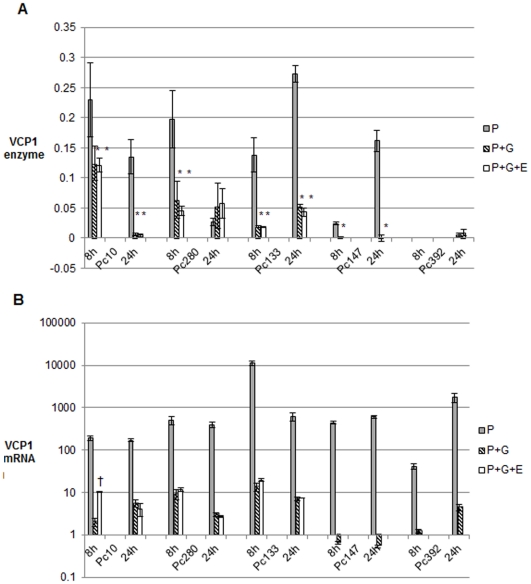
The effect of glucose and *M. incognita* eggs on VCP1 enzyme and mRNA production. Different isolates of *P. chlamydosporia* were grown in 0.01M potassium phosphate buffer pH 6.8 (P) with or without 2% glucose (G) and eggs (E, 1egg/µl). The effect of eggs was not tested on Pc147 and Pc392. Error bars denote standard errors of 3 biological replicates. In Fig. 2a, * denotes where the P+G and P+G+E means were significantly different (p<0.05) from the corresponding P mean; in Fig. 2b all of the P+G and P+G+E means were significantly different from their corresponding P mean (p<0.05). In Fig. 2b, † indicates where the P+G+E mean was significantly different (p<0.05) from the P+G mean. In Fig. 2a none of the P+G+E means was significantly different from the corresponding P+G mean.

In isolates Pc10, 133, 147 and 280, 2% (110 mM) glucose significantly repressed VCP1 enzyme levels (Fig. 2a) and mRNA levels (Fig. 2b) compared to the control at 8 h after transfer to the new medium. These effects were also seen at 24 h except for Pc280 where the enzyme levels were similarly low in both media. Levels of VCP1 enzyme inPc392 were very low at all time points to 24h, whether glucose was present or not, and there were no significant differences between treatments, so the effect of glucose on enzyme production was unclear. Real-time PCR data did however show that repression of VCP1 also occurred at 8 h and 24 h in the presence of glucose in this isolate. Lower concentrations of glucose (50 mM and 10 mM) were also shown to be effective at repression of VCP1 (enzyme and mRNA) in Pc10 and Pc280 and there was no significant difference between using 10 mM or 50 mM glucose (data not shown).

Adding *M. incognita eggs* (to isolates Pc10, 280 and 133) did not overcome the reduction in VCP1 enzyme or mRNA that occurred in the presence of glucose (Fig. 2). However in Pc10 at 8 h there was a small but statistically significant (p<0.05) increase in VCP1 mRNA in the presence of eggs.

### Ammonium Chloride Initially Represses, then Later Induces VCP1 Production

**Figure 3:**
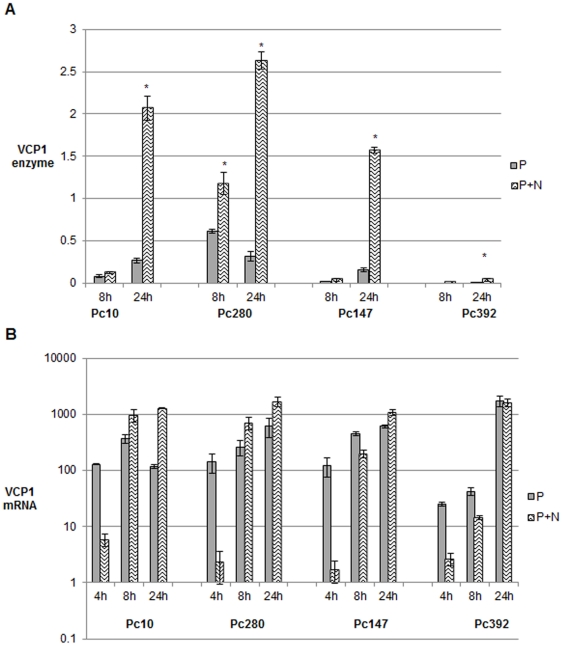
The effect of ammonium chloride on VCP1 enzyme and mRNA production. Different isolates of *P. chlamydosporia* were grown in P buffer with or without 200 mM ammonium chloride (N). Error bars denote standard errors of the biological replicates. In Fig. 3a, * denotes that the mean value for P+N is significantly different (p<0.05) from the corresponding P mean; in Fig. 3b all of the P+N means are significantly different from their corresponding P mean (p<0.05) except for that of Pc392 at 24h.

In isolates Pc10, Pc147, Pc280 and Pc392 there was a significant (p<0.05) increase in VCP1 (enzyme and mRNA [except Pc392]) levels 24 h after transfer to medium containing NH_4_Cl (Fig 3). However at 4 h the reverse was true; VCP1 mRNA levels were considerably reduced in the presence of ammonium chloride in these isolates (Fig. 3b). As VCP1 enzyme levels were very low in all isolates in all media at 4 h, it was not possible to draw any conclusions about enzyme levels at this time point (data not shown). At 8 h the effect differed depending on the isolate. In Pc10, Pc147 and Pc392 VCP1 enzyme levels were similar whether ammonium chloride was present or not but VCP1 mRNA levels in the presence of ammonium chloride were significantly (p<0.05) lower in Pc147 and 392 and higher (p<0.05) in Pc10. In Pc280 VCP1 enzyme and mRNA levels were both elevated in the presence of ammonium chloride at 8 h.

### VCP1 Production is Regulated by Ambient pH

**Figure 4:**
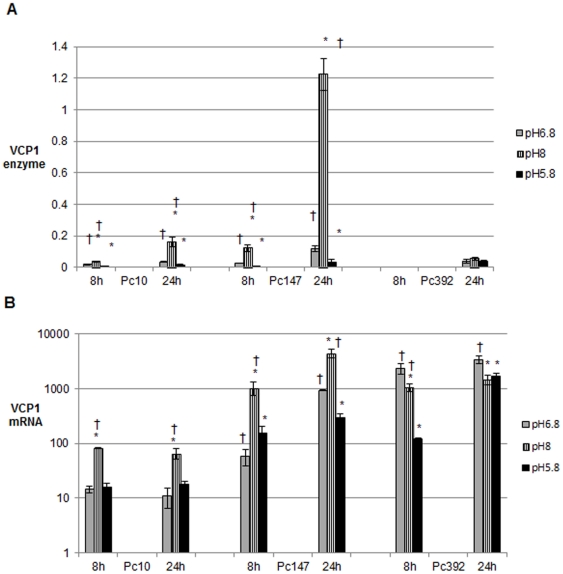
The effect of pH on VCP1 enzyme and mRNA production. Different isolates of *P. chlamydosporia* were grown in 0.01M potassium phosphate buffers at different pHs (pH 5.8, pH 6.8 and pH 8). Error bars denote standard errors of the biological replicates. An asterisk denotes that the mean value is significantly different (p<0.05) from the corresponding pH 6.8 mean and † denotes that the mean value is significantly different (p<0.05) from the corresponding pH 5.8 mean.

The effect of growth at pH 8.0, 6.8 and 5.8 on VCP1 levels was studied (Fig. 4). In Pc10 and Pc147 at 8 h and 24 h VCP1 (enzyme and mRNA) levels were significantly (p<0.05) higher in the pH 8 media, than in the other two media. In Pc392 enzyme levels were low in all three buffers and there were no significant differences (at the 5% level) between them. However VCP1 mRNA levels were significantly (p<0.05) higher when grown at pH 6.8 than at the other two pHs.

### Cryo-Scanning Electron Microscopy of *P. chlamydosporia* Interacting with *M. incognita* Eggs Reveals Damage Consistent with VCP1 Protease Attack

**Figure 5:**
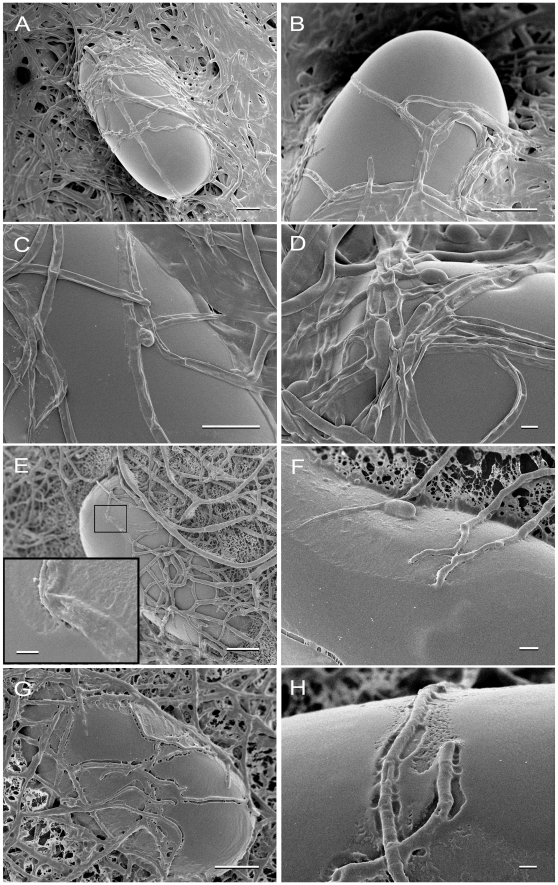
Cryo-SEM micrographs of Pc10 incubated with *M. incognita* eggs for 24h in different media. Isolate Pc10 was incubated with eggs for 24 h in phosphate buffer (P, Fig. 5A-B), P+2% glucose (Fig. 5C–D) or P+ 200 mM ammonium chloride (Fig. 5E–H). In Fig. 5A, B, C, E and G, bar = 10 µm, in Fig. 5D, E inset, F and H, bar = 1 µm.

Cryo-scanning electron microscopy (CSEM) was used to study the interaction of *P. chlamydosporia* on *Meloidogyne incognita* eggs (Fig. 5). This technique allows the examination of frozen, fully hydrated samples in their native state [16]. The preservation by rapid freezing in liquid nitrogen, avoids the removal or modification of tissue components such as waxes, biofilms and extracellular mucilage. It also prevents movement and separation of structures all of which are important in this study.

*P. chlamydosporia* isolate Pc10 was incubated with *M. incognita* eggs for 24 h as described for the VCP1 expression experiments, using 0.01M K phosphate buffer (P), or P containing either 2% glucose (P+G) or 200 mM ammonium chloride (P+N). The surface of intact undamaged eggs usually appears smooth and continuous when observed using Cryo-Scanning SEM. This smooth surface was observed in controls where eggs were incubated in the buffers but without fungus (data not shown). This was also the case where the eggs and fungus were grown in P or P+G (Fig. 5A–D), consistent with the idea that VCP1 levels had been too low by 24h to detect any damage to the outer proteinaceous vitelline membrane of the egg. However, in the P+N culture there was evidence that this layer had been damaged, consistent with VCP1 protease attack; in Fig. 5E and F extensive ‘rough’ patches could be seen on the egg surface surrounding the areas where hyphae were in contact with the egg, whereas areas further away remained smooth. Fig. 5G and H show that the hyphae have severely damaged the outer layer of the egg creating a ‘groove’ along their path of contact with the surface. There was no evidence of such egg damage by hyphae in the cultures that were grown in P or P+G (Fig. 5A–D).

## Discussion

*P. chlamydosporia* is a promising nematode biocontrol agent and a source of potentially valuable biochemical products including possible anti-microbial and anti-cancer agents [17], [18]. However, to date, with a few exceptions [19], [20], [21] there has been little work done to investigate the effect of environmental factors on gene expression in *P. chlamydosporia.* The alkaline serine protease VCP1 from this fungus is involved at the onset of the parasitic phase of its life cycle. It digests the outer proteinaceous vitelline membrane of nematode eggs facilitating penetration and infection. An understanding of how nutrients and other factors affect its expression is therefore critical for ensuring the efficacy of *P. chlamydosporia* as a biocontrol agent. Here we present new information on *vcp1* regulation and compare this with other subtilisin-like enzymes involved in the infection of nematode and insect hosts.

Many extracellular enzymes produced by fungi to degrade the outer cell layers of their hosts are regulated by carbon and nitrogen sources and pH [22]. Often this regulation is mediated by transcription factors which bind to DNA motifs upstream of the coding region of the gene. In our study, the upstream region of the subtilisin-like extracellular proteinase VCP1 of 30 *P. chlamydosporia* isolates was found to contain such motifs suggesting that it also would be subject to carbon, nitrogen, and pH regulation. All of the *P. chlamydosporia* isolates studied had highly conserved *vcp1* upstream regions, particularly with regard to these motifs. Studies of enzyme and mRNA expression confirmed that this gene was indeed subject to nutrient and pH regulation. The upstream region of one isolate Pc392, a *P. chlamydosporia* var. *catenulata* strain, was less well conserved (91–92% identity) than the other isolates (>97% identity). It also was found to be regulated by carbon and nitrogen and pH but some aspects of the regulation were different.

The presence of easily-metabolised carbon sources such as glucose result in the repression of various fungal genes needed to metabolise other carbon sources [23], [24]. In many ascomycete fungi this glucose repression is mediated by homologs of the products of the *creA* gene, studied most extensively in *A. nidulans*, but other genes e.g. *creB* and *creC* can also be involved [25], [26], [27], [28]. The upstream region of *vcp1* had previously been sequenced for *P. chlamydosporia* isolate Pc10 and positions of some of the putative carbon and nitrogen regulatory motifs determined [15] (Accession number AJ427460). However, subsequent analysis of this sequence revealed that the first 165nt were not derived from Pc10 but were an artefact from the linkers used in the cloning procedure. From nt 166 onwards the earlier Pc10 sequence is identical to our revised Pc10 sequence (Accession No. HE653897), to isolate 400 (the longest sequence, Accession No. HE653898) and six of the other isolates sequenced. Gene expression experiments monitoring both enzyme and mRNA of VCP1 confirmed that regulation by readily-metabolised carbon sources such as glucose did occur and that relatively low levels of glucose (10 mM) were sufficient to have an effect. It was not possible to overcome this repression by adding nematode eggs.

Carbon regulation of similar infection-related serine proteases occurs in other nematophagous and entomopathogenic fungi. The PR1 gene of the entomopathogen *M. anisopliae*, is subject to CREA-mediated carbon repression [29], [30]. The nematode trapping fungus *Arthrobotrys oligospora* produces a carbon-regulated cuticle-degrading serine protease (PII) that is an important pathogenicity factor [31], [32].

In many fungi the regulation of gene expression by nitrogen sources has been found to involve GATA transcription factors [33], [34], [35], [36]. The presence of GATA motifs in the upstream region of *vcp1* indicated that this gene also would be subject to regulation from nitrogen sources. However, whilst we demonstrated this to be the case, the effects were not exactly as predicted from studies of other fungi. Preferred nitrogen sources such as ammonium chloride have been shown to repress subtilisin-like serine proteases from other pathogens e.g. *Candida albicans* [37], *M. anisopliae* [29], *Arthrobotrys oligospora* [31] and *Clonostachys rosea* [38]. At 4 h after transfer to new medium, this was also the case for all *P. chlamydosporia* isolates tested, and at 8 h for Pc147 and Pc392. However by 24 h, ammonium chloride had significantly stimulated VCP1 expression. This could be because *P. chlamydosporia* behaves differently to the other fungi but it may also be that differences in the media (e.g. pH, presence/absence of carbon source) and protocols used (e.g. time of sampling) are responsible. The repression seen in the presence of ammonium chloride may only be a transitory effect that wears off over time. Alternatively, it is possible that ammonium chloride itself represses VCP1 production, but as the fungus continues to grow in a nutrient-deficient medium at neutral pH, it depletes NH_4_Cl and produces other nitrogen-containing metabolites from the ammonium that are inducers of VCP1. It is possible that different rates of C and N depletion, from the medium and the fungal internal stores, might be affecting the timing of responses by different isolates. However, we think it is unlikely that fungus grown with glucose or ammonium chloride has become starved for C or N during the time period monitored in these experiments (up to 24 hours); increases in fungal biomass were generally small in our experiments indicating that the fungus was in the early phases of its growth curve. Also in other filamentous fungi, the stationary phase was not usually observed until after 24 hours and often occurred much later than this [39]. It is clear however, that the differences in the physiological state of the fungus and nutrients available prior to and during its period of monitored growth can have a profound effect on gene expression.

The expression of *vcp1* was also affected by the pH of the medium in which *P. chlamydosporia* was grown. Many fungal extracellular enzymes are synthesised only at pH values in which they can function and complex genetic regulatory systems exist to ensure this [40]. Although there are several genes (seven in *A. nidulans*) involved in pH regulation, the zinc finger transcription factor PacC has been shown to play a major role in a range of fungi [40], [41], [42], [43]. This transcription factor mediates its effect by binding to a specific nucleotide motif (5′GCCARG) upstream of the genes which it regulates. The upstream region of the VCP1 gene of *P. chlamydosporia* isolates contains such motifs and it is likely that pH regulation of its expression is PacC-mediated. In *M. anisopliae* various extracellular proteolytic enzymes were shown to be regulated by pH, and the culture pH at which maximum activity of each enzyme was detected was close to its pH optimum [44]. This included several subtilisin-like Pr1 isoforms with pH optima around 8 that were produced only under alkaline conditions. Previous studies indicated that VCP1 activity is greatest at alkaline pH [8], [45], and our work using Pc10 and Pc147 showed that the expression of *vcp1* was higher when the culture was grown at pH 8 than at lower pHs. The expression of *vcp1* in the other isolate studied, Pc392, was higher when grown at pH 6.8 than at pH 8.0 or pH 5.8 and it had an extra PacC site in the upstream region that was not present in other isolates. However, Pc392 VCP1 enzyme assays performed at pH 8.0 and pH 7.0 gave very similar results (data not shown), so it does not appear that the Pc392 enzyme has a lower optimum pH for activity.

The exact composition and pH of the egg masses produced by *Meloidogyne* spp. is not known but in *Meloidogyne javanica* the gelatinous matrix is largely composed of low and high molecular weight glycoproteins that are rich in asparagine and glutamine [46]. Preliminary experiments were done to investigate the pH of egg masses; when egg masses (intact or broken) were placed in phenol red indicator (yellow at neutral pH), they turned red indicating that they are alkaline. However, pH measurements using a High Impedance Electrometer indicated the pH was just below neutral (around pH 6.5), with the egg interior being slightly more alkaline than the outside (personal communication, S. Smith and T. Miller, Rothamsted Research).

Regulation of infection-related genes by pH also occurs in other nematophagous and entomopathogenic fungi. In the nematophagous fungus *Clonostachys rosea* the PacC-mediated pH response has been shown to play an important role in pathogenesis [47]. Expression of the subtilisin-like extracellular protease *PrC* of *C. rosea* was upregulated in the presence of nematode cuticles but this only occurred under alkaline growth conditions [38]. Under acidic conditions, cuticles failed to stimulate the expression of PrC. Thus the fungus can down-regulate an infection related gene in conditions where the enzyme it encodes is ineffective. In the pine wilt nematode-trapping fungus *Monacrosporium megalosporium* the serine protease gene *spr1* was not transcribed at pH 4 whereas under alkaline conditions such as pH 8 and 9, the gene was transcribed well [48]. Some fungi not only have pathogenicity genes that are affected by pH, they can also actively increase or decrease the surrounding pH to optimise the host-pathogen interface for the appropriate virulence factor involved [49]. In *M. anisopliae,* pH regulates the expression of subtilisin proteases and *M. anisopliae* can regulate its ambient pH by production of ammonia [50].

Since Pc392 produced relatively low amounts of VCP1 enzyme under all conditions and time points tested, the effect of different media on enzyme production was not always clear for this isolate. In other studies, Pc392 also exhibited low enzyme levels under most conditions [19], [51]. This is intriguing, as Pc392 has a distinctive upstream sequence with a different regulatory motif profile from the other isolates. VCP1 mRNA levels were also low when compared to other isolates (data not shown), but due to the greater sensitivity of real-time PCR it was possible to monitor gene expression changes using this method. Pc392 contains exactly the same putative carbon regulatory motifs (CREB and CREA) in its upstream region as do the other isolates, and like them it shows repression of VCP1 when glucose is added. Two of the putative GATA sites found in other isolates are absent in Pc392, but despite this, it also shows *vcp1* repression during the first 4 h after transfer to ammonium chloride-containing medium. There are other putative GATA sites in all isolates including Pc392 that could be used as well or instead, so the absence of these two sites may not be important. However at 24h Pc392 differs in that there is no significant effect of ammonium chloride on VCP1 mRNA levels although as in other isolates VCP1 enzyme levels appear to be elevated. Pc392 has a different PacC motif profile from the other isolates and its optimum for VCP1 production is different (pH 6.8 rather than pH 8) from the other two isolates where effect of pH was studied (Pc10 and 147).

Earlier data from our group had indicated that the presence of nematode eggs stimulated VCP1 production; in a preliminary study (one experiment with two replicates), the addition of *M. incognita* eggs to Pc10 grown in mineral salts medium at 23°C had resulted in an approximately 4 fold increase in VCP1 enzyme production at 8h [45]. However in the current study there was little evidence that eggs had any effect on VCP1 production when either enzyme (released into the culture medium) or mRNA levels were measured. Although the protocols in the two studies were similar there were some differences that could account for this. In the earlier study the fungus had been starved of nutrients when the eggs were added, and the growth medium was un-buffered so it is possible that physiological or pH differences might have been responsible for the apparent stimulation by eggs. Differences in the condition of the eggs (the previous study used freeze-dried eggs) or the ratio of eggs to fungus used may also have been significant.

However, although it was not clear whether enzyme and mRNA levels in the culture as a whole were affected by the presence of eggs, there were other indications of egg-induced VCP1 production by the fungus. Microscopy studies using fluorescently labelled antibodies [8], [45] showed high levels of VCP1 localised to regions where the fungus was in contact with eggs. There was also some evidence from scanning electron microscopy for erosion of the egg surface where hyphae had been present [8]. Similarly in our study, electron micrographs of fungal-egg co-cultures (Fig. 5) indicated that the outer layer of the egg was altered in the vicinity of the fungal hyphae and this effect was only observed in media such as P+N where VCP1 levels were high (Fig. 5E-H) and not where levels were lower (in P [Fig. 5A–B] or P+G buffer [Fig. 5C–D]). Previous work has indicated that VCP1 is the major extracellular protease in *P. chlamydosporia* responsible for degrading the outer proteinaceous vitelline membrane [7], [8], [45]. It seems possible therefore that nematode eggs do stimulate VCP1 production, but only at a very localised level where the two are in close contact. This could explain why no major effect on VCP1 enzyme levels in the culture medium or mRNA levels in the fungus as a whole was seen when eggs were added; a large proportion of the fungal mycelium was not in close contact with the eggs (Fig. 5), therefore any stimulatory effect is likely to be diluted. The technical difficulty of producing sufficiently large numbers of *M. incognita* eggs for well-replicated studies precludes experiments with higher egg to fungus ratios. Also, when the fungus is grown in liquid cultures with eggs, it would not be able to adjust the microhabitat, for example the local pH at hyphal tips, which may be possible in solid substrates such as soil.

In different *Trichoderma* (*Hypocrea*) species many genes that encode proteases (particularly subtilisin-like serine proteases) are upregulated before and during contact with other fungi that they parasitise [52], [53], [54]. In entomopathogens enzymatic dissolution of the insect cuticle is often localised to the vicinity of fungal structures although extensive cuticle dissolution can also occur [55], [56]. In *Metarhizium anisopliae*, the expression of several extracellular proteolytic enzymes including Pr1 was increased when minimal medium was supplemented with insect cuticle [29], [44], [57]. This fungus is a facultative saprophyte with both soil-dwelling and insect pathogenic life-stages, and there were considerable differences in gene expression between growth in rich media, root exudates, insect cuticle and hemolymph [58], [59]. When fungus was transferred to bean root exudate media the alkaline serine protease Pr1A, which is closely related to VCP1, was one of the most abundant transcripts and it was 2.3X (24h)–3.3X (8h) more highly expressed in exudate than in rich medium; there were no data from nutrient-poor medium available for comparison. Colonization of the root surface by *P. chamydosporia* is closely linked to egg mass production and is thought to be related to changes in root exudation induced by the nematodes [60]. However, when we tested root exudates (buffered to pH 6.8) from either *M. incognita* -infected or non-infected tomato plants, these had a negligible effect on Pc10 VCP1 production (data not shown).

In most cases, corresponding VCP1 mRNA and enzyme levels showed similar trends, although differences between mRNA levels were generally considerably higher than between levels of enzyme activity. However, there were a few discrepancies. The enzyme assay depends on the protease being able to digest a particular sequence of amino acids in proteins. Therefore, theoretically there is a possibility that other, unknown enzymes, produced by the fungus might also have this capability. However, any enzyme that was only produced intracellularly, would not affect the assay, as culture medium is used as the source of the enzyme. In culture filtrates, VCP1 accounts for most of the protease activity of *P. chlamydosporia*, so the contribution of other enzymes to assays with this substrate would be minimal [15]. We think it is more likely that the few discrepancies that occur between mRNA levels and protease activity are related to the time delays between mRNA synthesis and production of enzyme and/or export into the medium. This could be influenced by post-transcriptional or post-translational modifications, degradation or inactivation of mRNA and/or enzyme. We know very little about these processes in *Pochonia*, and it is an area that warrants further study.

In conclusion, various factors were demonstrated to regulate the expression of the infection-related gene *vcp1* of *Pochonia chlamydosporia*. VCP1 production was repressed in glucose medium and most isolates produced more VCP1 at alkaline pH. However, whilst *vcp1* was repressed for a few hours in an ammonium chloride-containing medium, at later time points the enzyme was induced. Although the presence of nematode eggs did not appear to influence *vcp1* expression in the fungus as a whole, there was evidence of increased expression where the fungus was in close contact with the eggs. Since VCP1 is important in the initial stages of parasitism and the switch between the saprophytic and parasitic phase, these findings have implications for determining the optimal conditions in which *P. chlamydosporia* should be applied as a biocontrol agent. It seems clear that the presence of readily metabolisable carbon sources in the environment would not be conducive to pathogenicity of the fungus, and that alkaline conditions would favour egg-infection for most isolates. The situation with regard to ammonium chloride and other readily-metabolisable nitrogen sources, however, appears to be more complex and would depend on various other factors such as carbon levels and ambient pH. Also, whilst the carbon, nitrogen and pH conditions of the soil are important, it is possible that the micro-environment (rhizosphere and egg masses) surrounding the eggs may be of more relevance in determining biocontrol efficacy; VCP appears to be expressed most highly where the egg and fungus are in close proximity. Further work is needed to better understand the composition of the ecological niche in which *P. chlamydosporia* operates and the effects of different combinations of pH and carbon and nitrogen sources and ratios on *vcp1* expression and pathogenicity.
